# Nutritional Influences on Brown and Beige Adipocyte: Unraveling the Molecular Mechanisms and Metabolic Implications

**DOI:** 10.1002/fsn3.70613

**Published:** 2025-07-15

**Authors:** Yuqun Wang, Kexin Zhang, Chengxia Kan, Wenqiang Zhang, Xiaodong Sun, Lixin Li

**Affiliations:** ^1^ Department of Endocrinology and Metabolism, Shandong Provincial Key Medical and Health Discipline of Endocrinology and Laboratory of Endocrinology and Metabolic Diseases Clinical Research Center, Affiliated Hospital of Shandong Second Medical University Weifang China; ^2^ School of Public Health, Shandong Second Medical University Weifang China; ^3^ College of Health Professions, Central Michigan University Mount Pleasant Michigan USA

**Keywords:** brown adipose tissue, dietary intervention, metabolic disorders, nutrition, thermogenesis, uncoupling protein 1

## Abstract

Brown adipose tissue (BAT) and beige adipose tissue (beige fat) function as thermogenic organs, significantly influencing human metabolism through various mechanisms. Recent findings suggest that dietary nutrient supplementation targeting BAT offers innovative strategies for enhancing energy homeostasis and addressing metabolic diseases. Identifying the molecular mechanisms through which nutrition regulates the function of brown and beige adipocytes is essential for translating preclinical findings into clinical applications. This review initially delineates the physiological characteristics of BAT and elucidates the regulatory mechanisms underlying its metabolic functions and associated signaling pathways. Subsequently, we examine the impact of macronutrients, micronutrients, and nutritional stimulants on BAT functionality. Finally, we explore the therapeutic potential of refined dietary strategies as future approaches for managing metabolic disorders by targeting BAT. In conclusion, nutrient‐focused dietary strategies can enhance BAT function and activity, thereby contributing to the modulation of metabolic disorders and the optimization of metabolic health.

## Graphical abstract

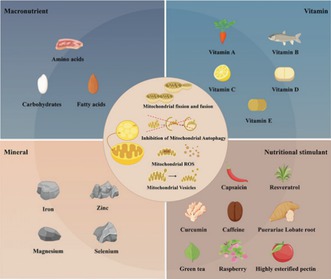

## 1 Introduction

Adipose tissue was recognized as an endocrine organ with essential metabolic functions in the 1980s (Cook et al. 1987). BAT acts as a key metabolic regulator, coordinating glucose, lipid, and amino acid homeostasis, thermogenesis, and the progression of metabolic disorders from obesity to cachexia (Auger and Kajimura 2023; Cohen and Kajimura 2021). Adipose tissue is categorized into white adipose tissue (WAT) and brown adipose tissue (BAT). WAT primarily stores energy and secretes hormones to maintain metabolic balance, characterized by multiple lipid droplets and few mitochondria (Scheja and Heeren 2019). In contrast, BAT, rich in both mitochondria and lipid droplets, functions as a thermogenic organ. Activated by sympathetic nerves in response to cold, BAT generates heat without shivering, constituting a key homeostatic nexus in coordinating thermal defense mechanisms with lipid mobilization dynamics (Hachemi and U‐Din 2023).

Recent studies underscore BAT's function in thermogenesis and energy homeostasis, revealing that various macronutrients and micronutrients can modulate BAT activity, presenting opportunities for dietary interventions that target BAT (Borcherding et al. 2022; Lu and Cao 2023; Yang et al. 2023). Plant‐derived compounds such as tea catechins, resveratrol, and capsaicinoids may stimulate BAT, potentially aiding in obesity management and regulating cardiometabolic disorders (Osuna‐Prieto et al. 2021). Understanding the molecular mechanisms by which these nutrients influence BAT could provide novel strategies for dietary optimization and supplementation to enhance BAT activity and improve disease outcomes (Yoneshiro et al. 2019). Therefore, this paper will focus on the molecular mechanisms by which nutrient factors affect BAT activity and their contribution in regulating metabolism through BAT. This paper also discusses some dietary components that were not previously addressed in any previous reviews.

## 2 Brown Adipose Tissue and Beige Fat

### 2.1 Origins, Structure, and Function of BAT

BAT, first described in 1551 by Conrad Gessner as the “hibernating gland,” is essential for thermogenesis (Gessner 1551). Initially recognized for its thermogenic properties, its facultative capacity in non‐shivering thermogenesis in rodents was demonstrated in 1978 (Foster and Frydman 1978). Cannon and Nedergaard (2004) later elucidated BAT's dual capacity in both thermoregulated and metabolically regulated thermogenesis, with the latter influenced by hypothalamic pathways. Active BAT promotes thermogenesis through enhanced glucose uptake and increased uncoupling protein 1 (UCP1) expression, especially in cold‐exposed individuals (Virtanen et al. 2009). Research by Cypess et al. (2009) and van Marken Lichtenbelt et al. (2009) in 2009 highlighted elevated BAT activity during hypothermia in adults, leading to further studies on transcriptomics, metabolomics, mitochondrial metabolism, and thermogenesis in BAT.

BAT, with its multilocular lipid droplets and mitochondria‐rich structure, generates heat via UCP1‐driven mitochondrial electron transfer, independent of ATP synthesis (Cohen and Kajimura 2021; Klingenspor et al. 2008). Beyond thermogenesis, BAT is critical for metabolic homeostasis, acting as a “glucose sink” and regulating glucose uptake (Carson et al. 2020). Bartelt et al. (2011) demonstrated that increased BAT glucose uptake in cold‐exposed obese mice improves insulin resistance and glucose tolerance. Cheng et al. (2021) discussed the potential of BAT browning agents in treating obesity and type 2 diabetes. BAT also secretes factors such as adiponectin, leptin, and fibroblast growth factor 21 (FGF21), which enhance insulin sensitivity, modulate metabolism, and support cell proliferation (Yang and Stanford 2022). In addition, its immunomodulatory effects are significant; for example, Moon et al. (2020) found that BAT from mice with collagen‐induced arthritis exhibited elevated phosphatidylinositol 3‐kinase/Akt signaling pathway (PI3K‐AKT) and interleukin 17 (IL‐17) levels compared to normal BAT, indicating potential benefits for autoimmune diseases.

In conclusion, BAT is essential for thermogenesis, energy metabolism, and overall metabolic health, with functions extending beyond mere heat production to include pleiotropic regulatory capacity in metabolic regulation and immune function. This multifunctionality of BAT provides valuable insights into its potential as a therapeutic target for metabolic disorders and autoimmune diseases.

### 2.2 Origins, Structure, and Function of Beige Fat

Early adipose tissue research focused on WAT and BAT, but with advances in research methods, beige fat has gained attention. Beige adipocytes primarily arise from preexisting WAT and can be induced by specific factors (Himms‐Hagen et al. 2000; Wu et al. 2012). Beige fat shares similarities with BAT in cellular structure and gene expression, including UCP1 and PGC1‐α (Harms and Seale 2013). UCP1‐driven mitochondrial uncoupling in beige adipocytes dissipates energy as heat, increasing energy expenditure and contributing to temperature regulation in response to cold (Ikeda et al. 2017; Kazak et al. 2015). Beige fat activity is also influenced by dietary factors, such as specific fatty acids (Abe et al. 2022; Okla et al. 2017; Sidossis and Kajimura 2015). However, in obesity and metabolic disorders, beige fat's thermogenic function is often impaired, leading to reduced energy expenditure and exacerbating conditions like insulin resistance (Czech 2020; Sidossis and Kajimura 2015). Therefore, activating beige fat's thermogenic function offers potential for obesity prevention and management. Given the similarities between beige fat and BAT, further investigation into their biological and therapeutic potential is warranted for obesity treatment and metabolic disease management.

### 2.3 Mechanisms of Mitochondrial Function in Facilitating Brown Adipocyte Thermogenesis

**FIGURE 1:**
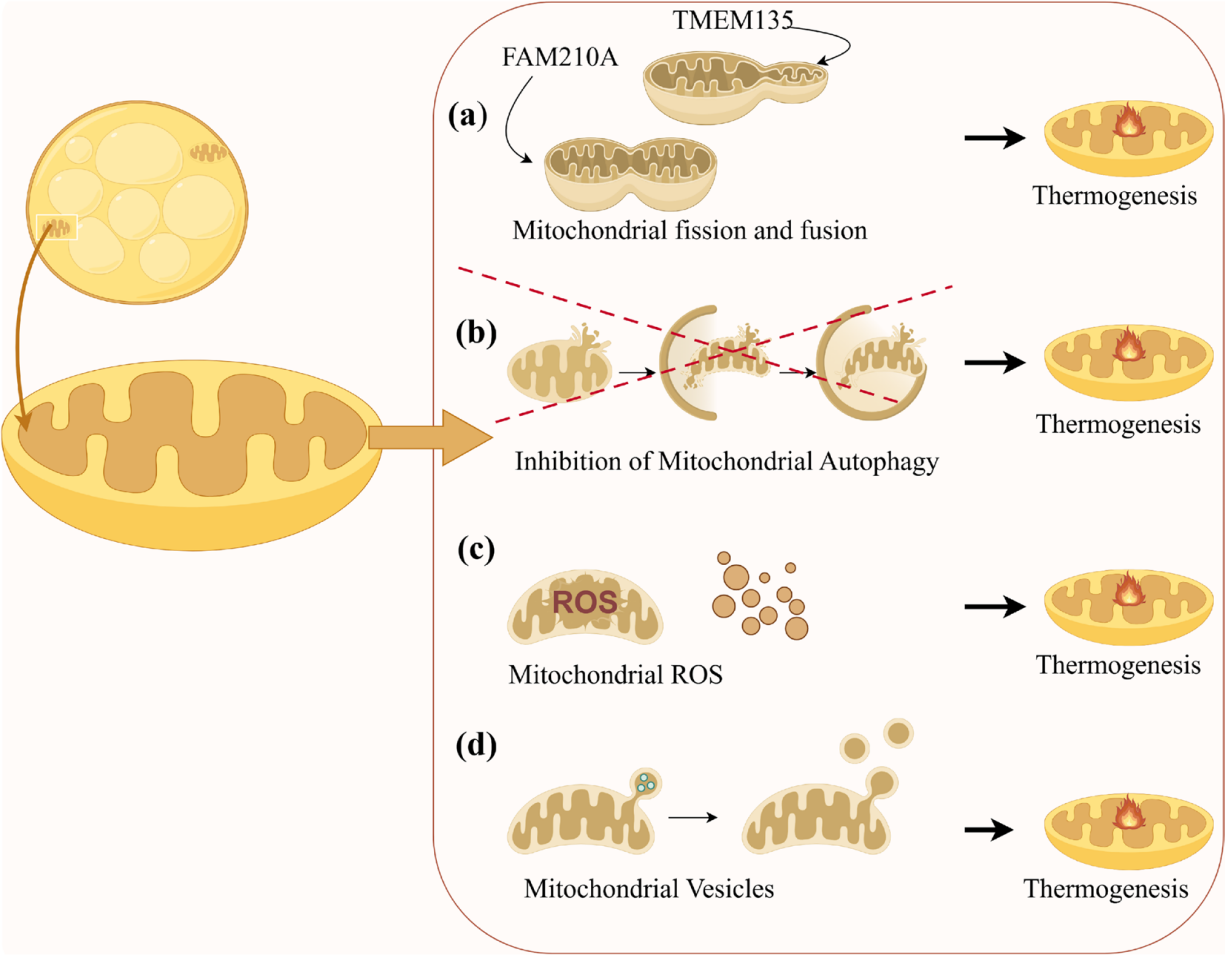
Mechanisms of mitochondria in BAT thermogenesis. (a) BAT enhances heat production through mitochondrial fission and remodeling mechanisms. (b) BAT enhances thermogenesis by inhibiting mitochondrial autophagy mechanisms. (c) BAT promotes the thermogenic function of UCP1 by elevating mitochondrial ROS levels. (d) BAT regulates adaptive thermogenesis through the release of mitochondria‐derived vesicles.

BAT is essential for non‐shivering heat production in mammals exposed to cold, primarily due to its abundant mitochondria (Porter et al. 2016) (Figure 1). The plasticity of mitochondria makes them a central focus in BAT research, as they support various thermogenesis pathways. Studies have identified multiple mechanisms through which mitochondria facilitate thermogenesis in BAT. One such mechanism is mitochondrial fission, regulated by transmembrane protein 135 (TMEM135), which highlights potential therapeutic targets for activating BAT (Hu et al. 2023) (Figure 1a). Another mechanism involves mitochondrial remodeling facilitated by a family with sequence similarity 210 member A (FAM210A), which enhances thermogenesis in cold environments by altering mitochondrial structure (Qiu et al. 2023) (Figure 1a). Additionally, inhibiting mitochondrial autophagy promotes thermogenesis (Figure 1b), whereas defective autophagy, such as through autophagy‐related 5 (Atg5) protein inhibition, leads to adipose dysfunction and metabolic disorders (Ko et al. 2021; Wu et al. 2019; Yau et al. 2021). Elevated levels of reactive oxygen species (ROS) also support UCP1‐dependent thermogenesis and increase overall energy expenditure (Chouchani et al. 2016) (Figure 1c). Furthermore, BAT regulates adaptive thermogenesis through the release of mitochondria‐derived vesicles (Ceci et al. 2022) (Figure 1d). In summary, BAT's reliance on mitochondrial functions is pivotal for thermogenesis and metabolic regulation. Consequently, research into the morphology, structure, and molecular mechanisms of mitochondria could significantly enhance our understanding of the biological functions of BAT.

### 2.4 Classic Molecular Cell Signaling Pathways Involved in BAT Thermogenesis

Several classical signaling pathways contribute to BAT thermogenesis, including the β3‐adrenergic receptor, the cAMP‐PKA pathway, the AMPK pathway, and the mTOR pathway, each serving as a critical regulator of thermogenic processes.

#### 2.4.1 β3‐Adrenergic Pathway

**FIGURE 2:**
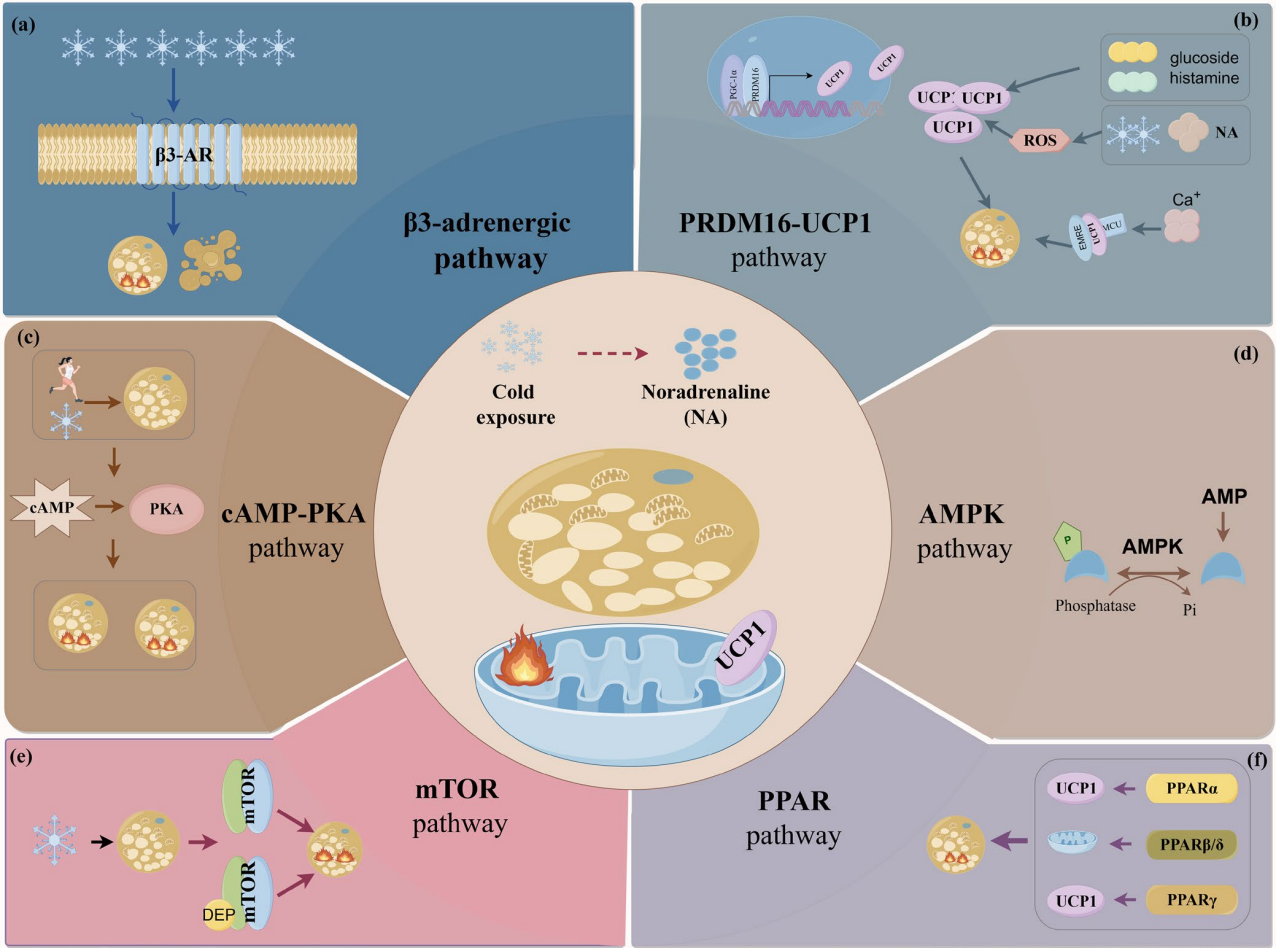
Classic molecular pathways involved in BAT thermogenesis. (a) The β‐adrenergic receptors (β‐AR) pathway is involved in adipocyte thermogenesis in BAT. (b) In BAT, UCP1, and PRDM16 act as key signaling pathways involved in the function of adipocytes. (c) The cyclic adenosine monophosphate‐protein kinase A (cAMP‐PKA) pathway is involved in adipocyte thermogenesis in BAT. (d) In BAT, adipocyte function involves the AMP‐activated protein kinase (AMPK) signaling pathway. (e) In BAT, adipocyte function involves the mammalian target of rapamycin (mTOR) signaling pathway. (f) In BAT, adipocyte function involves the peroxisome proliferator‐activated receptors (PPARs) signaling pathway.

β‐adrenergic receptors (β‐AR), particularly β3‐adrenergic receptors, have gained attention for their regulatory involvement in regulating thermogenesis in BAT (Wallukat 2002) (Figure 2a). Animal studies show that β‐AR‐mediated thermogenesis is vital for body temperature regulation in mammals and that β‐agonists can activate BAT and promote browning in adipose tissue (Oiwa et al. 2020; Wang et al. 2021). Combining β3‐agonists with antioxidants has shown promise in reducing inflammation in diet‐induced obesity (Abdul Sater et al. 2022). Innovative drug delivery methods, such as poly(propylene lactone‐glycolide copolymer acid) microspheres with β3‐agonist mirabegron, have successfully activated thermogenic adipocytes (Niu et al. 2024). Additionally, systemic administration of oxytocin and β3‐agonists can induce weight loss through enhanced BAT thermogenesis (Slattery et al. 2024). The significance of β3‐AR in regulating BAT thermogenesis in humans remains controversial (Lu, Jiang, et al. 2024). A recent study reveals that β3‐ARs are required to maintain the lipolytic and BAT thermogenic cellular machine in human brown and beige adipocytes; however, that study used polyclonal primary human preadipocytes (Cero et al. 2021). In human clinical trials, pharmacological interventions targeting the β3‐AR failed to produce optimal outcomes in terms of lipolysis and thermogenesis within BAT (Blondin et al. 2020). Although the exact function of β3‐AR in human BAT remains unclear, the classical nature of this pathway warrants further investigation to elucidate its underlying mechanism.

#### 2.4.2 PRDM16‐UCP1 Pathway

The molecular cell signaling pathways in BAT are essential for understanding metabolic regulation and energy expenditure. Key pathways involve UCP1 and PRDM16, essential for thermogenesis and brown fat development. UCP1 is essential for heat production in brown adipocytes. Research has found that cyanidin‐3‐O‐glucoside enhances BAT's thermogenic capacity by modulating UCP1, and histamine mediates β3‐adrenergic agonists' effects on adipogenesis by regulating UCP1 (Feng et al. 2021; Han et al. 2021). Further studies identified that the mitochondrial calcium uniporter (MCU) binds with UCP1, forming the MCU‐EMRE‐UCP1 complex, which promotes thermogenesis (Xue et al. 2022). Additionally, ROS are involved in BAT's thermogenic function via the UCP1 pathway during cold exposure or norepinephrine stimulation (Oo et al. 2022). PRDM16 is a vital transcription factor for maintaining BAT's differentiation, morphology, and function. There is also a positive correlation between UCP1 and PRDM16 expression, highlighting their integrated synergistic functions in BAT (Gu et al. 2023). The PRDM16–UCP1 signaling pathway serves as a central regulatory mechanism in the differentiation, development, and functional maintenance of BAT (Figure 2b).

#### 2.4.3 cAMP‐PKA Pathway

The cyclic adenosine monophosphate‐protein kinase A (cAMP‐PKA) signaling axis centrally regulates cellular functions and metabolism via extracellular signal transduction (Figure 2c). In adipocytes, activation of the β3‐AR/cAMP/PKA pathway promotes lipolysis and thermogenesis, particularly in response to cold exposure or thermogenic stimuli (Park et al. 2021; Zhou et al. 2020). Physical exercise also enhances thermogenesis in BAT via this pathway (Yao et al. 2024). Recent studies have investigated that drugs and nutrients can modulate BAT function through the cAMP‐PKA signaling cascade (Ding et al. 2023; Lu et al. 2021). Overall, the cAMP‐PKA pathway is a primary regulator of BAT thermogenesis, energy expenditure, cell differentiation, and metabolic processes.

#### 2.4.4 AMPK Pathway

AMP‐activated protein kinase (AMPK) is an essential nutrient sensor that maintains cellular energy homeostasis (Figure 2d). Prolonged cold exposure enhances AMPK activity in BAT, primarily influencing chronic thermogenesis rather than acute non‐shivering thermogenesis (Mulligan et al. 2007). Huang et al. (Zhang et al. 2022) first suggested that intestinal AMPK regulates BAT thermogenesis, impacting systemic energy balance and glucose homeostasis. Recent studies have shown that various substances, including drugs and nutrients, can modulate BAT function through the AMPK pathway (Pacifici et al. 2023; Xu et al. 2021; Zhang et al. 2024). Furthermore, evidence suggests that PGC‐1α, a key modulator of uncoupled respiration in brown adipocytes, also facilitates enhanced thermogenesis via the activation of the AMPK signaling cascade (Chen et al. 2021; Pettersson‐Klein et al. 2018). To summarize, AMPK is vital in regulating BAT activity, modulating energy metabolism in response to cold exposure, and interacting with sympathetic nerve activity, thereby influencing energy homeostasis and thermogenesis.

#### 2.4.5 mTOR Pathway

Mammalian target of rapamycin (mTOR), a major effector molecule of rapamycin, is central to the regulation of cell growth and metabolic processes (Figure 2e). Within adipose tissue, the mTOR signaling pathway significantly influences the browning process and thermogenic capacity of the tissue (Mao and Zhang 2018). Furthermore, both mTOR complexes have been associated with UCP1 expression levels and the thermogenic potential of BAT (Castro et al. 2021; Festuccia 2024). DEP‐domain‐containing mTOR‐interacting protein (DEPTOR) is highly expressed in brown adipocytes, with its expression increasing during brown adipocyte development and adaptation to cold environments (Colas et al. 2023). Overall, the mTOR signaling pathway orchestrates spatiotemporal coordination of BAT ontogeny, functional maturation, and catabolic‐anabolic balance, mechanistically bridging progenitor differentiation, mitochondrial plasticity, and whitening‐to‐browning transitions.

#### 2.4.6 Peroxisome Proliferator‐Activated Receptors Pathway

Peroxisome proliferator‐activated receptors (PPARs) are a family of nuclear receptors significantly involved in lipid metabolism, primarily through the transcriptional regulation of target genes (Figure 2f). This receptor family comprises three distinct isoforms: PPARα, PPARβ/δ, and PPARγ. In BAT, activation of PPARα has been shown to upregulate UCP1 expression and augment mitochondrial metabolism (Li, Wu, et al. 2023). Animal studies have confirmed that PPARα agonists promote enhanced adaptive thermogenesis in functional brown adipocytes (Santana‐Oliveira et al. 2022). Additionally, PPARβ/δ acts as a key regulator of adaptive thermogenesis in BAT by coordinating fatty acid oxidation and mitochondrial biogenesis (Palomer et al. 2018). Notably, PPARγ has been recognized as a master regulator of BAT, functioning as the primary orchestrator of brown adipocyte differentiation and metabolic programming (Koppen and Kalkhoven 2010). A study revealed that acetylation of PPARγ promotes the whitening of BAT and impairs its functionality (He et al. 2023). Recent studies have demonstrated that PPAR agonists (PPARα and PPARγ) reduce body weight, ameliorate insulin resistance, induce the browning of beige adipocytes, enhance both UCP1‐dependent and independent thermogenesis, and improve mitochondrial metabolism in murine models (Miranda et al. 2024). Ultimately, PPARs regulate fatty acid oxidation, mitochondrial function, and thermogenesis in BAT through distinct mechanisms, maintaining body temperature. Synthesizing the findings, several key molecular pathways regulate brown and beige fat differentiation and function, offering therapeutic potential for metabolic disorders. However, the current understanding of how these cell signaling pathways regulate the function and development of brown and beige fat in humans remains limited.

## 3 Nutrition Effect on BAT Function and Activity

### 3.1 Macronutrients Influence BAT Development and Activity

Macronutrients, including amino acids, carbohydrates, and fatty acids, are fundamental to human metabolic processes. Recent studies have revealed the substantial impact of macronutrients on BAT function and whole‐body energy homeostasis. Therefore, this study provides key insights into the potential of dietary interventions to enhance BAT activity, potentially unveiling new therapeutic strategies to improve metabolic health and address metabolic disorders (Table 1).

**TABLE 1:** Nutrient effects on BAT.

Nutrient	Structure	Mechanisms	References
Macronutrients			
Amino acid			
Valine	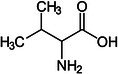	Thermogenesis	Westerterp‐Plantenga et al. (1999)
Leucine	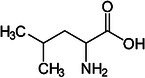	Thermogenesis Energy metabolism	Ho (2018), Westerterp‐Plantenga et al. (1999)
L‐arginine	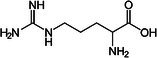	BAT development	Ma et al. (2017)
Carbohydrate			
High‐carbohydrate diet	—	Inhibition of BAT activity	Waldhart et al. (2021)
Ketogenic diet	“Fat”: “combined protein and carbohydrates” = 4:1	Thermogenesis Energy metabolism	Ahmad, Seo, and Jang (2024), Li et al. (2024), Qiu, Zhang, et al. (2024), Srivastava et al. (2013)
Fatty acid			
Omega‐3 fatty acid		Thermogenesis Energy metabolism	Kalupahana et al. (2020), Wang et al. (2024)
ALA	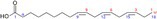	Energy metabolism	Wang et al. (2024)
EPA	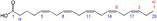	Lipid metabolism	Kalupahana et al. (2020), Kim et al. (2015), Soni et al. (2019), Wang et al. (2024)
DHA	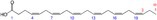	Lipid metabolism	Kalupahana et al. (2020), Kim et al. (2015), Soni et al. (2019), Wang et al. (2024)
5‐HEPE	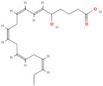	Promotion of WAT browning BAT activity	Zong et al. (2023)
Omega‐6 fatty acid		BAT activity	Maliszewska et al. (2022)
CLA	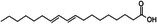	Promotion of WAT browning	Shen et al. (2013)
Trans‐10, cis‐12	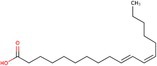	Inhibition of BAT activity Thermogenesis	Rodríguez et al. (2002), Shen et al. (2015)
Trans‐11, cis‐9	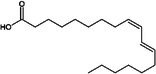	Promotion of BAT activity Thermogenesis	Rodríguez et al. (2002), Shen et al. (2015)
Oleic acid	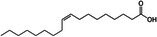	BAT activity	Oi‐Kano et al. (2017)
Micronutrients			
Vitamins			
Vitamin D	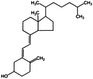	BAT activity Energy metabolism	Cheung et al. (2020), Mukai and Kusudo (2023), Zhao and Qin (2022)
Vitamin A	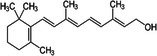	Thermogenesis	Fenzl et al. (2020), Herz and Kiefer (2020), Kumar et al. (1999), Nie et al. (2017)
Vitamin C	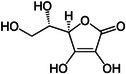	Thermogenesis	Behrens and Madère (1981), Lee et al. (2022), Mory et al. (1983)
Vitamin B5	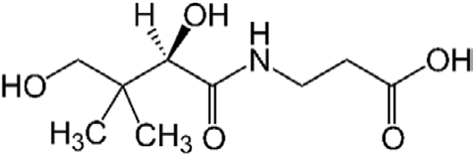	Thermogenesis	Zhou et al. (2022)
Vitamin B1	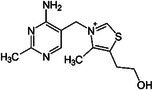	Thermogenesis	Takeda and Dai (2024)
Vitamin B2	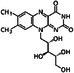	Thermogenesis	Takeda and Dai (2024)
Vitamin E	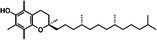	Antioxidant capacity	Bolin et al. (2024)
Minerals			
Iron	_26_Fe	Mitochondrial function	Lu, Guo, et al. (2024), Mai et al. (2024), Moreno‐Navarrete and Fernández‐Real (2024), Nazari et al. (2022), Qiu, Kan, et al. (2024), Yook et al. (2021)
Zinc	_30_Zn	Thermogenesis	Ahmad, Shaju, et al. (2024), Jiang et al. (2023), Kajimura et al. (2008), Sanders and Tisdale (2004)
Magnesium	_12_Mg	Thermogenesis	Al Alawi et al. (2018), Beavis and Garlid (1990), Kurstjens et al. (2018), Madaris et al. (2023)
Selenium	_34_Se	Thermogenesis	Jedrychowski et al. (2020), Kieliszek and Błażejak (2013), Li, Cheng, et al. (2023), Ruswandi et al. (2024), Shimada et al. (2022)
Nutritional stimulants			
Capsaicin	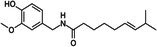	Thermogenesis Energy metabolism	Gavva et al. (2007), Harb et al. (2023), Irandoost et al. (2021), Liang et al. (2023), Perez et al. (2022), Wen et al. (2022)
Resveratrol	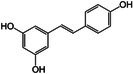	Thermogenesis BAT activity	Abra and Assis (2020), Andrade et al. (2019), Culum and Yurekli (2020), Kim et al. (2019), Milton‐Laskíbar et al. (2020)
Curcumin	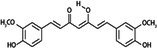	Thermogenesis Energy metabolism Lipid metabolism	Lone et al. (2016), Santos et al. (2023), Song et al. (2018), Teixé‐Roig et al. (2023), Zhao et al. (2021)
Caffeine	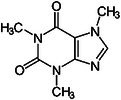	Thermogenesis Energy metabolism Lipid metabolism	Graneri et al. (2021), Martins et al. (2023), Pérez et al. (2021), Van Schaik et al. (2021, 2022), Velickovic et al. (2019), Yoneshiro et al. (2017)
Green tea		Thermogenesis Energy metabolism Lipid metabolism	Li et al. (2021), Sousa‐Filho et al. (2020), Zhao et al. (2022)
Raspberry		BAT activity Energy metabolism Lipid metabolism	Attia et al. (2019), Leu et al. (2018), Zou et al. (2018)
Puerariae lobata root extracts		BAT activity	Ahmad Khan and Ahmad (2019), Buhlmann et al. (2019), Kamiya et al. (2012), Yang et al. (2019)
Prebiotic high‐esterified pectin and its postbiotic acetate		Thermogenesis energy metabolism	García‐Carrizo et al. (2020), Gibson et al. (2017), Muñoz‐Almagro et al. (2021)

#### 3.1.1 Effects of Amino Acids

Amino acids, such as valine and leucine, are gaining recognition for their functional contribution in supporting thermogenic adipocytes (Westerterp‐Plantenga et al. 1999). When incorporated into dietary proteins, these amino acids can influence energy homeostasis and BAT activity, potentially enhancing thermogenesis and reducing body weight (Ho 2018). For instance, research has shown that L‐arginine stimulates the growth of brown adipocyte precursor cells via the mTOR signaling pathway, suggesting arginine supplementation may promote BAT development in fetal lambs (Ma et al. 2017). Similarly, Park et al. (2023) demonstrated that BAT regulates nitrogen uptake and fuel oxidation through amino acid consumption. These findings collectively indicate that specific amino acids serve as pivotal metabolic regulators in modulating BAT activity and overall energy balance. Recent animal studies have further underscored the significant impact of dietary amino acids on adipose tissue, revealing that supplementation with these nutrients can markedly decrease WAT and enhance BAT thermogenesis, thereby effectively mitigating obesity (Corsetti et al. 2022). These studies emphasize the potential benefits of tailored dietary strategies for optimizing metabolic health and the development of targeted nutritional approaches to enhance energy balance. Further research is essential to investigate these mechanisms and validate these effects in humans.

#### 3.1.2 Effect of Carbohydrate and Ketogenic Diet on BAT Activity

Carbohydrates act as metabolic gatekeepers, modulating BAT thermogenesis and systemic homeostasis. Low‐protein diets with fixed carbohydrate content can promote hyperphagia and increase energy expenditure through sympathetic activation (Zapata et al. 2019). However, excessive carbohydrate intake is associated with mitochondrial dysfunction in BAT, impairing mitochondrial integrity and electron transport chain efficiency (Waldhart et al. 2021).

The ketogenic diet (KD), characterized by minimal carbohydrate, moderate protein, and high fat intake (Ahmad, Seo, and Jang 2024), has been widely used for weight loss, sparking research into its effects on metabolism and adipose tissue (Li et al. 2024; Qiu, Kan, et al. 2024). One study reveals that a KD can increase BAT mitochondrial size and UCP1 protein levels (Srivastava et al. 2013). Another study found that only supraphysiological concentrations of β‐hydroxybutyrate (β‐HB) induce adipocyte browning. Surprisingly, physiological concentrations of β‐HB downregulate UCP1 expression and do not induce adipocyte browning (de Oliveira Caminhotto et al. 2019).

These findings highlight the complex interplay between dietary carbohydrates, KD, and BAT metabolism and thermogenesis. The impact of KD on the function of brown and beige fat in humans remains unknown.

#### 3.1.3 Influences of Fatty Acids on Energy Homeostasis Within BAT

Fatty acids are essential macronutrients, with omega‐6 and omega‐3 fatty acids being particularly important. Omega‐6 fatty acids are linked to BAT activity, with lower intakes correlating with detectable BAT activity (Maliszewska et al. 2022). Omega‐3 fatty acids, including alpha‐linolenic acid, eicosapentaenoic acid (EPA), and docosahexaenoic acid (DHA), have antioxidant properties and are vital for growth and development (Wang et al. 2024). A study showed that 5‐hydroxyeicosapentaenoic acid (5‐HEPE), an omega‐3 unsaturated fatty acid metabolite, activates BAT and induces WAT browning by upregulating UCP1, Prdm16, and PGC1α genes through the AMPK/PGC1α pathway (Zong et al. 2023). These fatty acids can reduce inflammation in WAT, enhance energy metabolism, and stimulate thermogenesis in BAT (Kalupahana et al. 2020). Supplementation with EPA and DHA in a high‐fat diet has been shown to decrease lipid accumulation in adipose tissue and enhance lipid metabolism by inducing UCP1 expression in brown and beige adipocytes, primarily through sympathetic activation (Kim et al. 2015; Soni et al. 2019).

Research indicates that a higher omega‐6/omega‐3 ratio is associated with obesity and negatively affects BAT function, thereby increasing obesity risk. Increasing omega‐3 intake or converting omega‐6 to omega‐3 may enhance BAT function and improve lipid metabolism (Simopoulos 2016). Conjugated linoleic acid (CLA), another polyunsaturated fatty acid found in meat and dairy, has been extensively studied for its regulatory impact on lipid metabolism (Shen et al. 2013). CLA exists in several isomers, including cis‐9, trans‐11 CLA (CLA‐9), and trans‐10, cis‐12 CLA (CLA‐10). These isomers have distinct effects: trans‐10, cis‐12 CLA reduces BAT mass and thermogenic capacity, while cis‐9, trans‐11 CLA enhances UCP1 expression and BAT thermogenesis (Rodríguez et al. 2002; Shen et al. 2015). Oleic acid, a monounsaturated fatty acid found in olive oil, works as an essential metabolic modulator in BAT thermogenesis and energy expenditure by increasing UCP1 expression in BAT (Oi‐Kano et al. 2017).

Overall, different fatty acids influence various metabolic processes and energy homeostasis within BAT. The future human study should focus on understanding the specific mechanisms and effects of different fatty acids, which will enable us to better tailor dietary interventions as therapeutic strategies to improve obesity and metabolic disorders.

#### 3.1.4 The Influence of Fasting on BAT Function

Intermittent fasting, a flexible dietary regimen, offers benefits for weight management, cardiovascular health, and metabolic function, encompassing both rhythmic and non‐rhythmic fasting patterns (Hofer et al. 2022). Intermittent fasting has also been explored for its potential to aid weight loss, improve metabolic health, and treat autoimmune diseases (Barati et al. 2023). Early research examined the impact of starvation and refeeding on triacylglycerol/fatty acid substrate cycling in BAT, providing a foundation for understanding fasting's effects on BAT (Mercer and Williamson 1988). Other research has shown that chronic cold exposure and every‐other‐day fasting (EODF) can induce weight loss through changes in body temperature and metabolism, highlighting fasting's impact on thermogenesis and BAT activity (Xu et al. 2023). EODF has also been shown to stimulate beige fat development through modification of gut microbiota composition and upregulation of monocarboxylate transporter 1 expression in beige adipocytes (Li et al. 2017). A retrospective analysis of a human study observed that prolonged fasting is a relatively safe dietary regimen, yielding short‐term clinical weight loss (Pietzner et al. 2024). However, its efficacy in achieving sustained improvements in metabolic markers remains uncertain (Ezpeleta et al. 2024). Thus, intermittent fasting influences BAT activity and thermogenesis through various mechanisms, including the differentiation of beige adipocytes and regulation of thermogenic genes. Further human clinical trials are needed to identify fasting's impact on BAT activity.

### 3.2 Micronutrients Influence BAT Biology

Micronutrients, including essential vitamins and minerals, are crucial even in small amounts. We investigate the effects of specific micronutrients on BAT (Table 1).

#### 3.2.1 Vitamins Influences BAT Function

Vitamins, essential compounds sourced from fruits, vegetables, and meat, function as indispensable mediators in metabolism and immune regulation by safeguarding cells and organs and being stored in the body (Stevens 2021). Recent studies have highlighted the impact of vitamins on BAT function.

##### 3.2.1.1 Vitamin D

Vitamin D is an important class of fat‐soluble steroid derivatives, consisting primarily of vitamin D2 (ergocalciferol) and vitamin D3 (cholecalciferol). Vitamin D3 reduces PPARγ, PGC‐1α, and UCP1 expression while regulating lipid metabolism via the PI3K/Akt/mTOR/p53 pathway (Zhao and Qin 2022). However, 1,25‐(OH)2D3 (calcitriol), an active metabolite of vitamin D, enhances UCP1, PRDM16, and Pgc1‐α expression within physiological ranges, supporting its BAT function and early brown adipocyte differentiation (Mukai and Kusudo 2023). Lu and Cao (2023) underscored the essential regulatory axis of the Vitamin D receptor (VDR). While VDR activation can suppress brown adipocyte differentiation and thermogenic gene expression, the effects are context‐ and dose‐dependent. Further animal studies demonstrated that vitamin D supplementation corrected the aberrant expression of molecules associated with adipose tissue browning (e.g., UCP1), resulting in weight loss and improved energy balance in mice (Cheung et al. 2020). In summary, vitamin D regulates BAT development and function primarily through VDR, with effects that vary depending on physiological context.

##### 3.2.1.2 Vitamin A

Vitamin A, a fat‐soluble vitamin, consists of two main classes: retinoids, including retinol, its metabolites, and synthetic analogs; and pro‐vitamin A carotenoids, plant‐derived precursors converted to retinol in the body. Vitamin A influences BAT function. Kumar et al. (1999) found that dietary vitamin A supplementation elevated UCP1 gene expression in the BAT of rats without changing β3‐AR gene expression, suggesting that vitamin A promotes UCP1 gene expression to regulate energy homeostasis. Herz and Kiefer (2020) highlighted the transcriptional contribution of vitamin A and the retinoid axis in brown fat function, emphasizing the importance of retinoids in adipose tissue thermogenesis. Fenzl et al. (2020) elucidated the mechanistic basis of intact vitamin A transport in cold‐stimulated adipocyte thermogenic programming, revealing the physiological necessity of retinoid trafficking for environmental adaptation responses. Furthermore, a study has indicated that selective activation of the retinoid X receptor (RXR) may offer a promising therapeutic strategy for modulating brown and beige fat function in vivo (Nie et al. 2017). Thus, vitamin A promotes brown fat function.

##### 3.2.1.3 Vitamin C

Vitamin C is a water‐soluble vitamin that is vital to human health. As early as the last century, scholars found that increased levels of ascorbic acid were found in the BAT of rats chronically exposed to cold (Behrens and Madère 1981; Mory et al. 1983). Lee et al. (2022) observed that vitamin C levels in BAT increased in response to cold exposure. Subsequent animal studies delineated the regulatory circuitry linking senescence marker protein 30 (SMP30)‐mediated vitamin C biosynthesis to PPARα/FGF21‐driven thermogenic programming, unveiling novel signaling properties of ascorbate in BAT functional adaptation (Lee et al. 2022). Overall, the current understanding of the effects of vitamin C on BAT and associated cell signaling pathways is relatively limited. Further investigation should focus on the underlying mechanism of action of vitamin C on BAT function with a particular emphasis on research involving human subjects.

##### 3.2.1.4 Vitamin B and Vitamin E

Vitamin B5, a water‐soluble vitamin found in a wide range of foods, has attracted interest due to its anti‐obesity effects, which are mediated through mechanisms involving BAT (Zhao et al. 2024). Research indicates that vitamin B5 significantly upregulates the expression of UCP1 in BAT. Through this mechanism, vitamin B5 may increase energy expenditure, reduce adiposity, and improve glucose homeostasis (Zhou et al. 2022). In addition to vitamin B5, vitamin B1 and vitamin B2 are also closely associated with BAT metabolic function (Takeda and Dai 2024). As essential coenzymes in energy metabolism, vitamin B1 and vitamin B2 may enhance BAT thermogenesis by supporting mitochondrial function and facilitating fatty acid oxidation (Ismail et al. 2020).

Additionally, vitamin E, an essential fat‐soluble antioxidant, has a major impact on protecting cellular structures from oxidative damage induced by free radicals (Niki 2015). It has also been shown to attenuate inflammatory responses in BAT by suppressing the production and secretion of pro‐inflammatory cytokines, thereby contributing to the preservation of BAT function under metabolic stress. Chronic and excessive exposure to glucocorticoids has been shown to induce the whitening of BAT, a process frequently associated with heightened inflammatory signaling (Bolin et al. 2024). However, owing to its antioxidant capacity, vitamin E may ameliorate these detrimental effects and promote the functional integrity of BAT (Bolin et al. 2024).

#### 3.2.2 Effects of Minerals on BAT Function and Thermogenesis

In addition to vitamins, minerals may also affect BAT, although this area has been studied less extensively.

##### 3.2.2.1 Iron

Iron is fundamental to energy metabolism by supporting ATP synthesis and the electron transport chain (ETC). Disruption of iron homeostasis, marked by elevated serum ferritin and excessive iron accumulation in tissues like the liver, adipose, and skeletal muscle, exacerbates adipose dysfunction (Moreno‐Navarrete and Fernández‐Real 2024). Mitochondrial biogenesis and function are dependent on iron (Mai et al. 2024). Studies show that excess iron in adipose tissue during browning, induced by β3‐AR activation, is linked to increased mitochondrial biosynthesis and respiration (Yook et al. 2021). Disrupting adipose iron homeostasis in mice enhances mitochondrial respiration and adipokine expression in BAT (Lu, Guo, et al. 2024). Iron deficiency impacts the production of beige fat, whereas iron chelation enhances beige fat differentiation and metabolic activity (Nazari et al. 2022). Additionally, elevated transferrin receptor levels in BAT of overweight individuals suggest its implication in obesity by regulating BAT (Qiu, Zhang, et al. 2024). Taken together, these studies underscore the pivotal involvement of iron in beige fat development, although this has not yet been proven in humans.

##### 3.2.2.2 Zinc

Zinc, an essential micronutrient, supports zinc‐dependent metalloenzymes and overall health (Ahmad, Shaju, et al. 2024).

Zinc‐alpha2‐glycoprotein (ZAG), a zinc‐binding protein, directly influences UCP1 expression in BAT to regulate thermogenesis, while PRDM16, a zinc‐finger protein, governs BAT determination by promoting brown adipocyte‐specific gene expression and repressing white adipocyte‐specific genes (Kajimura et al. 2008; Sanders and Tisdale 2004). Recent studies show that zinc ions enhance sympathetic innervation and thermogenesis in both BAT and subcutaneous WAT in male mice (Jiang et al. 2023).

##### 3.2.2.3 Magnesium

Magnesium, the fourth most abundant cation in the body, is central to functioning as a cofactor in over 300 enzymatic reactions (Al Alawi et al. 2018). As early as the last century, scientists found that elimination of the Mg^2+^ matrix activated K^+^‐H^+^ exchange activity in BAT mitochondria (Beavis and Garlid 1990). Studies suggest that magnesium deficiency may help combat diet‐induced obesity and metabolic disorders by activating metabolic pathways, increasing BAT UCP1 mRNA expression, and raising body temperature (Kurstjens et al. 2018; Madaris et al. 2023).

##### 3.2.2.4 Selenium

Selenium is a metallic element of atomic number 34 with antioxidant properties and involvement in lipid metabolism (Kieliszek and Błażejak 2013). Shimada et al. (2022) and Ruswandi et al. (2024) investigated the regulatory dynamics of selenium in BAT lipid metabolism, suggesting that selenium supplementation prevents adipose cell dysfunction and augments the bioenergetic potency of BAT triiodothyronine in thermogenesis. Notably, a study introduced selenium‐rich green tea polysaccharide from Ziyang, which increased the expression of thermogenic markers UCP1, PGC‐1α, and CIDEA in BAT, thereby promoting thermogenesis and mitigating obesity (Li, Cheng, et al. 2023). Additionally, Jedrychowski et al. (2020) developed a mass spectrometry method to track selenium incorporation into proteins and proposed that dietary selenium supplementation boosts UCP1 levels, enhances energy expenditure in thermogenic adipose tissue, and helps prevent obesity.

### 3.3 Nutritional Stimulants Influence BAT Biology

Nutritional stimulants exert a profound influence on human health, with research progressing from examining the risks associated with inadvertent stimulant consumption to investigating the potential of appetite stimulants in managing medical conditions. As early as 2004, van der Merwe and Grobbelaar (2004) underscored the dangers of unintentional intake of supplements containing stimulants such as ephedrine and caffeine. Subsequent studies have identified novel therapeutic approaches, including appetite stimulants, for addressing cachexia in patients with chronic obstructive pulmonary disease (COPD) (King et al. 2008). Furthermore, research has elucidated the effects of nutritional stimulants on lipid metabolism and metabolic disorders (Seoane‐Collazo et al. 2019). We also aim to analyze the individual nutrient extracts with single active ingredients (e.g., resveratrol) as well as mixtures (e.g., green tea) and the potential for inadvertent consumption of nutrients containing bioactive stimulatory compounds on BAT biology. Analyzing the impact of these stimulants on BAT may provide new insights for optimizing metabolic health through dietary and lifestyle interventions (Table 1).

#### 3.3.1 Capsaicin and Capsinoids Modulate BAT Activity

Capsaicinoids, primarily found in chili peppers, exhibit anti‐inflammatory and antioxidant effects, improve glucose homeostasis, and regulate lipid metabolism (Liang et al. 2023). A meta‐analysis revealed that capsaicin or capsaicinoids significantly increase resting metabolic rate, energy expenditure, and fat oxidation while decreasing the respiratory quotient and carbohydrate oxidation in humans (Irandoost et al. 2021). This effect is mediated through their action on the transient receptor potential vanilloid 1 (TRPV1) receptor, which is involved in thermoregulation and lipid metabolism (Gavva et al. 2007). Furthermore, a randomized controlled trial employing Mongolian gerbils revealed that capsaicin injections enhance thermogenic capacity through TRPV1 activation, positioning this ion channel as a pivotal mediator of behavioral thermoregulation pathways (Wen et al. 2022).

Dietary capsaicin supplementation alone has been shown to enhance BAT energy expenditure and substrate utilization, thereby inhibiting obesity. A systematic review (Perez et al. 2022) of six human studies assessed capsaicin's ability to activate the TRPV1 calcium channel, indirectly stimulating BAT and increasing UCP1 expression. Among these, three clinical trials demonstrated capsaicin's mechanistic involvement in boosting energy expenditure and reducing body weight, while the remaining studies revealed its effects on BAT thermogenesis. Capsaicinoids may emerge as effective nutritional agonists for weight control and obesity suppression, particularly when combined with physical activity or dietary strategies (Harb et al. 2023). Collectively, these studies highlight the significant translational potential of capsaicin and capsinoids in modulating BAT activity and energy expenditure through the TRPV1 receptor, suggesting potential targets and promising dietary strategies for addressing obesity and related metabolic diseases.

#### 3.3.2 Resveratrol Modulates BAT Function and Stimulates BAT Thermogenesis

Resveratrol, a non‐flavonoid polyphenol found in wine and grape juice, has garnered considerable research interest for its effects on adipose tissue, particularly BAT. Studies have demonstrated that resveratrol can induce white fat browning. Building on this, research has shown that resveratrol interacts with adrenomedullin, inhibiting angiogenesis in WAT while promoting it in BAT (Abra and Assis 2020; Kim et al. 2019). This dual action enhances energy expenditure and contributes to obesity reduction (Culum and Yurekli 2020). Administration of resveratrol increased thermogenesis and is associated with the upregulation of PRDM16, UCP1, and PGC1α in human studies (Andrade et al. 2019). Further supporting these findings, investigations into resveratrol and its derivative, pterostilbene, indicate that these compounds significantly stimulate BAT thermogenesis and WAT browning (Milton‐Laskíbar et al. 2020).

Taken together, these studies underscore the importance of resveratrol in enhancing BAT function, promoting thermogenesis, and inducing WAT browning. These findings suggest resveratrol's potential as a therapeutic agent for managing obesity and related metabolic disorders.

#### 3.3.3 Curcumin Improves BAT Function

Curcumin, a lipophilic polyphenol derived from turmeric, is implicated in cellular inflammation, apoptosis, and oxidative stress. Clinical trials have demonstrated that curcumin significantly enhances the effectiveness of various established drugs for immune system disorders, metabolic disorders, and inflammatory diseases. Curcumin facilitates lipolysis and inhibits adipogenesis, potentially benefiting metabolic health (Lone et al. 2016). Importantly, it boosts the expression of brown adipocyte‐specific genes, induces the browning of white adipocytes, and enhances mitochondrial biogenesis (Zhao et al. 2021). Studies show that curcumin supplementation increases UCP1 expression via both PPAR‐dependent and independent pathways, leading to higher energy expenditure and improved regulation of body temperature in response to cold stimuli in mice (Song et al. 2018). Curcumin supplementation enhances metabolic responses in adipose tissue by increasing mRNA expression of thermogenesis markers (UCP1 and PRDM16) (Santos et al. 2023). Encapsulating curcumin in an emulsion with medium‐chain triglycerides (MCT) boosts its distribution in BAT, improving its effectiveness in preventing obesity and metabolic disorders (Teixé‐Roig et al. 2023). Overall, curcumin supplementation may positively impact BAT function and metabolic health, although further research is needed to clarify these mechanisms and assess curcumin's potential as a treatment for obesity and metabolic disorders in human subjects.

#### 3.3.4 Caffeine Increases BAT Thermogenesis

Caffeine, increasingly consumed in energy drinks by adolescents and young adults, can contribute to metabolic syndrome and affect the cardiovascular, endocrine‐metabolic, and central nervous systems with long‐term use (Graneri et al. 2021). However, research indicates that caffeine can stimulate calorie release from brown fat, potentially aiding in the management of obesity and diabetes (Velickovic et al. 2019). Dietary caffeine supplementation has been shown to enhance metabolic homeostasis by boosting metabolism.

Low doses of caffeine can increase BAT thermogenesis without adverse cardiovascular effects, and caffeine activates orexin‐positive neurons in the lateral hypothalamus, which is involved in BAT thermogenesis (Van Schaik et al. 2021). The paradoxical effects of caffeine on peripheral adenosine receptors suggest that central mechanisms may underlie caffeine‐induced BAT thermogenesis and increased energy expenditure. Caffeine increases BAT temperature in healthy adult males (Van Schaik et al. 2022). Orally ingested tea catechin combined with caffeine acutely increases energy expenditure by enhancing BAT activity and chronically elevates non‐shivering cold‐induced thermogenesis (CIT) in humans (Yoneshiro et al. 2017).

In physically active men, those with high BAT activity experienced greater energy expenditure following caffeine supplementation (Pérez et al. 2021). Additionally, caffeine supplementation in mice promoted adipose tissue remodeling, reduced inflammation, and improved metabolic profiles related to obesity (Martins et al. 2023). These studies suggest that caffeine can activate BAT thermogenesis and enhance energy expenditure, particularly in physically active individuals with high BAT activity.

#### 3.3.5 Puerariae Lobata Root Extracts Influence BAT Activity

Herbal medicines are widely used, with 60% of the global population relying on them and 80% of people in developing countries depending on them for daily health needs (Ahmad Khan and Ahmad 2019). Recent research has revealed the antidiabetic, antioxidant, and insulin resistance‐alleviating properties of Pueraria Mirifica and its extracts. Puerariae lobata root extracts regulate metabolic homeostasis and improve insulin sensitivity through multiple mechanisms (Yang et al. 2019).

The extracts exhibit anti‐obesity effects in both humans and in mice fed with a high‐fat diet (Kamiya et al. 2012). The extract induces weight loss and improvement of glucose metabolism through stimulating BAT activity and the formation of beige fat in inguinal adipose tissue (Buhlmann et al. 2019). These studies underscore the potential of Puerariae Lobata root extracts in regulating BAT activity and enhancing metabolic health. Further research is needed to explore the cell signaling pathways involved and clarify the mechanisms by which these extracts influence BAT function and metabolic homeostasis.

#### 3.3.6 Green Tea Promotes BAT Production

Green tea, the most popular beverage globally after water, contains a range of pharmacologically active compounds, including tea polyphenols, catechins, and alkaloids (Zhao et al. 2022). As a dietary stimulant, green tea promotes BAT production, enhances BAT thermogenesis, and supports mitochondrial metabolism, aiding in anti‐obesity efforts. Research indicates that green tea increases lipolysis and induces the browning of subcutaneous WAT in mice through β3‐adrenergic receptor activation (Sousa‐Filho et al. 2020). Additionally, green tea extracts influence adipocyte differentiation and metabolism, suggesting that these components may combat obesity by activating BAT (Li et al. 2021).

Studies have shown that green tea extracts boost mitochondrial metabolism in brown adipocytes, presenting a promising therapeutic strategy for obesity and related metabolic disorders (Im et al. 2022). Collectively, these findings suggest that green tea and its components significantly influence adipose tissue metabolism, particularly by activating thermogenesis and browning in BAT. Further research is needed to fully explore the therapeutic potential of green tea for obesity and metabolic disorders.

#### 3.3.7 Raspberry Influences BAT Development

Raspberries, rich in polyphenols and derivatives, are gaining attention for their potential to promote brown adipogenesis, reduce adipose inflammation, modulate lipid metabolism, and improve insulin resistance (Attia et al. 2019). Raspberry ketone increases mitochondrial biogenesis and browning‐specific proteins, including PRDM16, PGC‐1α, and UCP1; induces the browning of WAT; increases BAT formation; and inhibits autophagy (Leu et al. 2018). Raspberries also promote the development of brown and beige adipocytes in mice fed a high‐fat diet through activating AMPKα, which enhances WAT browning and BAT activity, resulting in increased energy expenditure and fatty acid oxidation (Zou et al. 2018). Overall, raspberries and their bioactive compounds, such as raspberry ketones and polyphenols, have the potential for modulating adipose tissue metabolism, promoting the browning of white adipocytes, and enhancing BAT activity. These findings highlight the potential of raspberries as a natural ingredient for metabolic health and fat reduction. Further research is needed to understand the mechanisms behind their effects on thermogenesis and adipose tissue metabolism.

#### 3.3.8 Prebiotic High‐Esterified Pectin

High‐esterified pectin (HEP), a soluble prebiotic found in fruits and vegetables, regulates glucose homeostasis, lipid metabolism, inflammation, and body weight (Muñoz‐Almagro et al. 2021). Recent animal studies show that HEP enhances energy expenditure and metabolic homeostasis by promoting thermogenesis in BAT and browning of WAT (García‐Carrizo et al. 2020; Gibson et al. 2017). Interestingly, prebiotics that affect the host microbiota are a promising research focus. HEP also improves gut microbiota, increasing acetate production, which regulates BAT thermogenesis, fatty acid conversion, and adipokine production (García‐Carrizo et al. 2020). Further research is needed to explore how HEP and acetate affect BAT activity, particularly through mechanisms involving UCP1 expression and mitochondrial biogenesis.

## 4 Concluding Remarks

**FIGURE 3:**
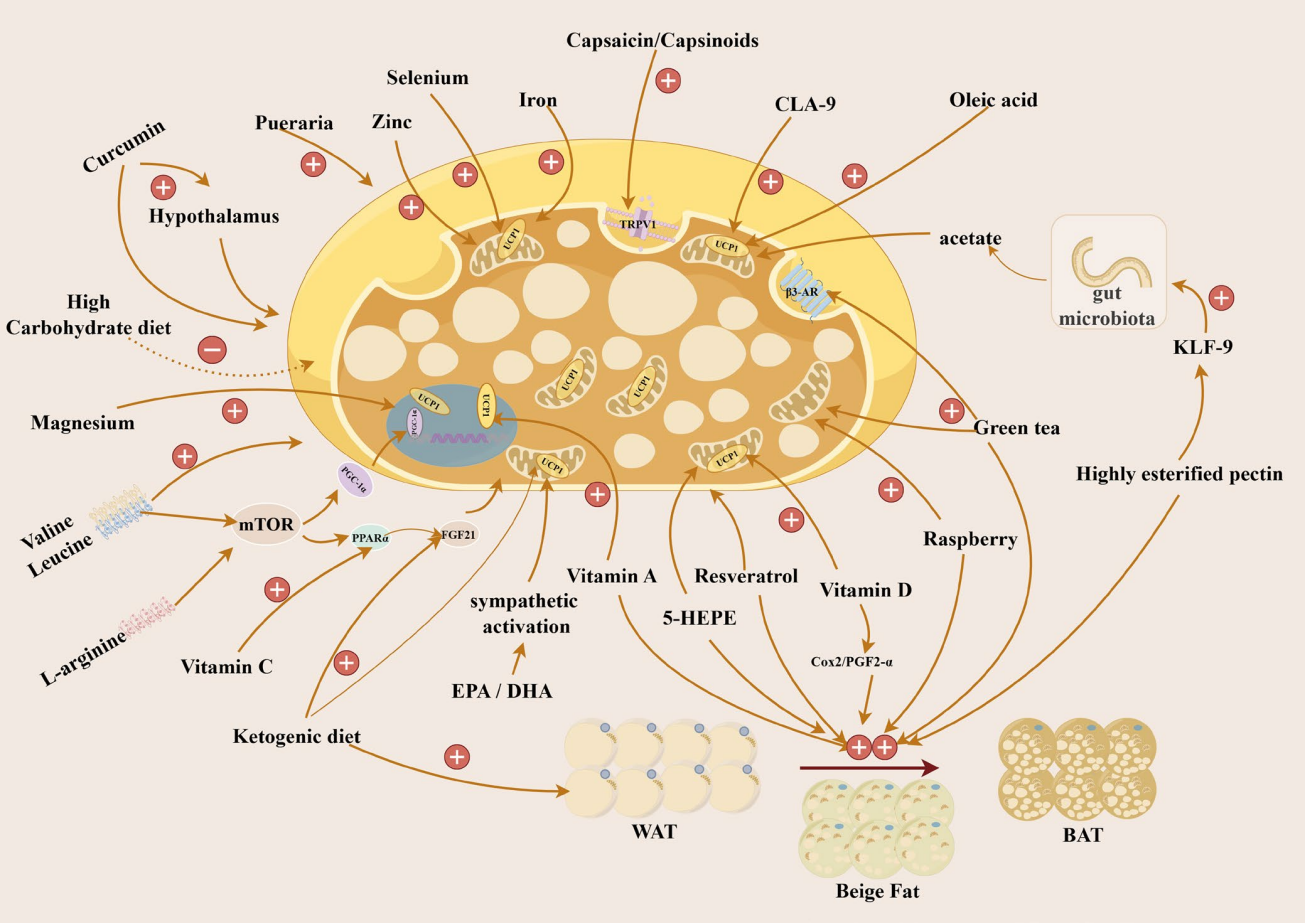
Effects of macronutrients, micronutrients, and nutritional stimulants on BAT. Here, solid red arrows indicate stimulation and dashed red arrows indicate inhibition. 5‐HEPE, 5‐hydroxyeicosapentaenoic acid; BAT, brown adipose tissue; CLA‐9, *cis*‐9, *trans*‐11 CLA, conjugated linoleic acid 9; COX2, cyclooxygenase‐2; DHA, docosahexaenoic acid; EPA, eicosapentaenoic acid; FGF21, fibroblast growth factor 21; KLF‐9, Kruppel‐like factor 9; mTOR, mammalian target of rapamycin; PGC‐1α, peroxisome proliferator‐activated receptor gamma coactivator 1 alpha; PGF2α, prostaglandin F2α; PPARα, peroxisome proliferator‐activated receptor alpha; UCP1, uncoupling protein 1; WAT, white adipose tissue.

BAT has emerged as a pivotal player in thermogenesis and metabolic regulation, offering a promising target for addressing metabolic disorders (Figure 3). This review explores the various aspects of BAT in energy homeostasis and glucose metabolism. Its unique thermogenic properties, mediated by UCP1, and its potential as a “glucose sink” underscore its significance in maintaining metabolic health. Nutritional factors, including specific macronutrients, micronutrients, and plant‐derived compounds such as amino acids, fatty acids, and vitamins, have shown potential in modulating BAT function, presenting opportunities for dietary interventions. Additionally, emerging evidence on fasting and dietary stimulants like capsaicin, resveratrol, curcumin, green tea, caffeine, and raspberry compounds further highlights the influence of nutrition in regulating BAT activity. As chronic and metabolic diseases rise, it is fundamental to develop tailored dietary interventions for patients with comorbidities, ensuring that restrictive diets do not worsen the primary condition. However, this review predominantly relies on animal and in vitro studies, with limited clinical trials in humans. The scarcity of human studies has hindered a deeper understanding of the specific adipose tissue types affected and the underlying mechanisms, limiting the clinical application of these findings. Future research should focus on elucidating the molecular pathways by which nutrients influence BAT, developing targeted dietary and pharmacological interventions, and conducting translational studies to validate these findings in human populations. By integrating insights from molecular biology, nutrition science, and clinical research, we can develop innovative strategies to enhance BAT function, ultimately improving metabolic health and combating obesity and related disorders.

## Author Contributions

**Yuqun Wang:** conceptualization (equal), data curation (equal), methodology (equal), writing – original draft (equal). **Kexin Zhang:** conceptualization (equal), data curation (equal), methodology (equal), writing – original draft (equal). **Chengxia Kan:** data curation (equal), investigation (equal). **Wenqiang Zhang:** data curation (equal), investigation (equal). **Xiaodong Sun:** conceptualization (equal), data curation (equal), funding acquisition (equal), project administration (equal), supervision (equal), validation (equal), writing – review and editing (equal). **Lixin Li:** conceptualization (equal), data curation (equal), project administration (equal), supervision (equal), validation (equal), writing – review and editing (equal).

## Ethics Statement

The authors have nothing to report.

## Conflicts of Interest

The authors declare no conflicts of interest.

## Data Availability

The authors have nothing to report.
